# Inhibition of Stearoyl-CoA Desaturase 1 Expression Induces CHOP-Dependent Cell Death in Human Cancer Cells

**DOI:** 10.1371/journal.pone.0014363

**Published:** 2010-12-16

**Authors:** Mélaine Minville-Walz, Anne-Sophie Pierre, Laurent Pichon, Sandrine Bellenger, Cécile Fèvre, Jérôme Bellenger, Christian Tessier, Michel Narce, Mickaël Rialland

**Affiliations:** Université de Bourgogne, Centre de Recherche INSERM «Lipides, Nutrition, Cancer» UMR866, Dijon, France; Texas A&M University, United States of America

## Abstract

**Background:**

Cancer cells present a sustained de novo fatty acid synthesis with an increase of saturated and monounsaturated fatty acid (MUFA) production. This change in fatty acid metabolism is associated with overexpression of stearoyl-CoA desaturase 1 (Scd1), which catalyses the transformation of saturated fatty acids into monounsaturated fatty acids (e.g., oleic acid). Several reports demonstrated that inhibition of Scd1 led to the blocking of proliferation and induction of apoptosis in cancer cells. Nevertheless, mechanisms of cell death activation remain to be better understood.

**Principal Findings:**

In this study, we demonstrated that Scd1 extinction by siRNA triggered abolition of de novo MUFA synthesis in cancer and non-cancer cells. Scd1 inhibition-activated cell death was only observed in cancer cells with induction of caspase 3 activity and PARP-cleavage. Exogenous supplementation with oleic acid did not reverse the Scd1 ablation-mediated cell death. In addition, Scd1 depletion induced unfolded protein response (UPR) hallmarks such as Xbp1 mRNA splicing, phosphorylation of eIF2α and increase of CHOP expression. However, the chaperone GRP78 expression, another UPR hallmark, was not affected by Scd1 knockdown in these cancer cells indicating a peculiar UPR activation. Finally, we showed that CHOP induction participated to cell death activation by Scd1 extinction. Indeed, overexpression of dominant negative CHOP construct and extinction of CHOP partially restored viability in Scd1-depleted cancer cells.

**Conclusion:**

These results suggest that inhibition of de novo MUFA synthesis by Scd1 extinction could be a promising anti-cancer target by inducing cell death through UPR and CHOP activation.

## Introduction

Cancer cells exhibit metabolism alterations characterised by increased glycolysis and lipogenesis [1], [2]. Active proliferating cancer cells present not only quantitative changes in *de novo* lipid biosynthesis but also modifications of lipid membrane composition affecting membrane fluidity, signal transduction and gene expression [3], [4]. Changes in lipid membrane composition are observed in a wide variety of cancers, mainly characterised by saturated (SFA) and monounsaturated fatty acid (MUFA) accumulation which appears less due to increased uptake of SFA and MUFA than to exacerbated endogenous fatty acids synthesis, irrespective of adequate lipid nutritional supply [5], [6], [7], [8], [9], [10], [11]. These modifications of SFA and MUFA content are associated with the modulation of the expression and activity of lipogenic enzymes. Thus, overexpression of acetyl Co-A carboxylase α and fatty acid synthase, involved in the first steps of fatty acid biosynthesis, were described in various cancers [12], [13], [14], [15], [16], [17].

Increased MUFA content could be also due to an up-regulation of stearoyl Co-A desaturase (Scd, delta-9 desaturase) expression, the rate-limiting enzyme of MUFA synthesis. Indeed, Scd catalyzes the introduction of a double bond between carbons 9 and 10 of several saturated fatty acids such as palmitic (16∶0) and stearic (18∶0) acids to yield palmitoleic (16∶1) and oleic (18∶1) acids, respectively. This endoplasmic reticulum resident enzyme exists under two isoforms in humans, Scd1 and Scd5 [18]. Scd1 is found in almost all tissues with a major expression in liver while Scd5 expression is restricted to pancreas and brain. Scd1 expression, correlated with MUFA content, is increased in hepatocellular adenoma, colonic and oesophageal carcinoma, as well as in genetically- and chemically-induced tumors [19], [20], [21]. For prostate cancer, two studies present contradictory results on Scd1 expression level [22], [23]. Thus, Scd1 expression can be related to carcinogenesis processes involving alteration of proliferation/apoptosis balance. Indeed, Scd1 over-expressing cells present a growth advantage while scd1 knock-down leads to slower rates of cell proliferation and cell death *in vivo* and *in vitro* [24], [25], [26], [27]. The mechanism of cell death observed in Scd1-deficient lung cancer cells seems to involve the modification of a SFA/MUFA ratio that triggers inhibition of the Akt pathway and activation of the AMPK pathway [24], [28]. Indeed, in absence of Scd1, the SFA content increases which alleviates Akt activation normally obtained by MUFA (e.g. oleic acid) for sustaining cell proliferation and survival [29]. Furthermore, different cancer cells lacking Scd1 activity reduce *de novo* lipogenesis through activation of the AMPK pathway [22], [24]. The alteration of lipid production in Scd1-deficient cells mainly concerns a reduction of phospholipid biosynthesis, which triggers cellular stress and expression of the apoptosis-related protein C/EBP homologous protein (CHOP/GADD153) [26], [27], [30], [31]. CHOP belongs to a peculiar stress pathway named endoplasmic reticulum (ER) stress that may induce apoptosis.

ER stress is triggered by different stress conditions such as alterations in post-translational protein status and lipid synthesis, hypoxia, disruption of calcium homeostasis and nutrient deprivation, and leads to the activation of an adaptive program, known as the Unfolded Protein Response (UPR), to re-establish equilibrium [32]. Activation of the canonical UPR engages three distinct concerted signalling branches mediated by ER membrane anchored sensors: RNA-dependent protein kinase (PKR)-like ER kinase (PERK), activating transcription factor 6 (ATF6) and inositol requiring enzyme 1α (IRE1α) [33]. In stressed cells, the chaperone protein GRP78 dissociates from UPR sensors PERK, ATF6 and IRE1α leading to their activation to first alleviate ER stress. PERK phosphorylates the eukaryotic translation initiation factor (eIF)2α, thereby inhibiting global protein synthesis. Active ATF6 translocates to the Golgi and is cleaved from the membrane by site-1 and -2 proteases. Then cleaved ATF6 localises to the nucleus and induces transcription of Xbp1 and ER chaperones such as GRP78. IRE1α disposes of an endoribonuclease activity that alternatively splices the Xbp1 mRNA (sXbp1), which is translated into an active transcription factor. However, in a severe or prolonged stress, the UPR can trigger pro-apoptotic signals through activation of the transcription factor CHOP, which acts to repress the bcl-2 gene expression, thus down-regulating anti-apoptotic Bcl-2 protein and rendering cells sensitive to the pro-apoptotic effects of BH3-only proteins [34], [35], [36].

While these data clearly support the involvement of Scd1 as a central regulator of lipogenesis in cancer cells, the link between Scd1 and the induction of ER stress and cell death in cancer cells remains to be better understood. In this study, we were consequently interested in seeking for UPR induction in cancer cells lacking Scd1 expression and in investigating the role of this stress pathway on the cancer cell viability during Scd1 extinction. We demonstrated that Scd1 depletion in cancer cells activated UPR markers and induced cancer cell death with no effect on non cancer cell viability. Moreover, we evidenced that CHOP participated to Scd1-mediated cell death.

## Results

### Efficient inhibition of Scd1 expression and suppression of *de novo* MUFA synthesis

**Figure 1:**
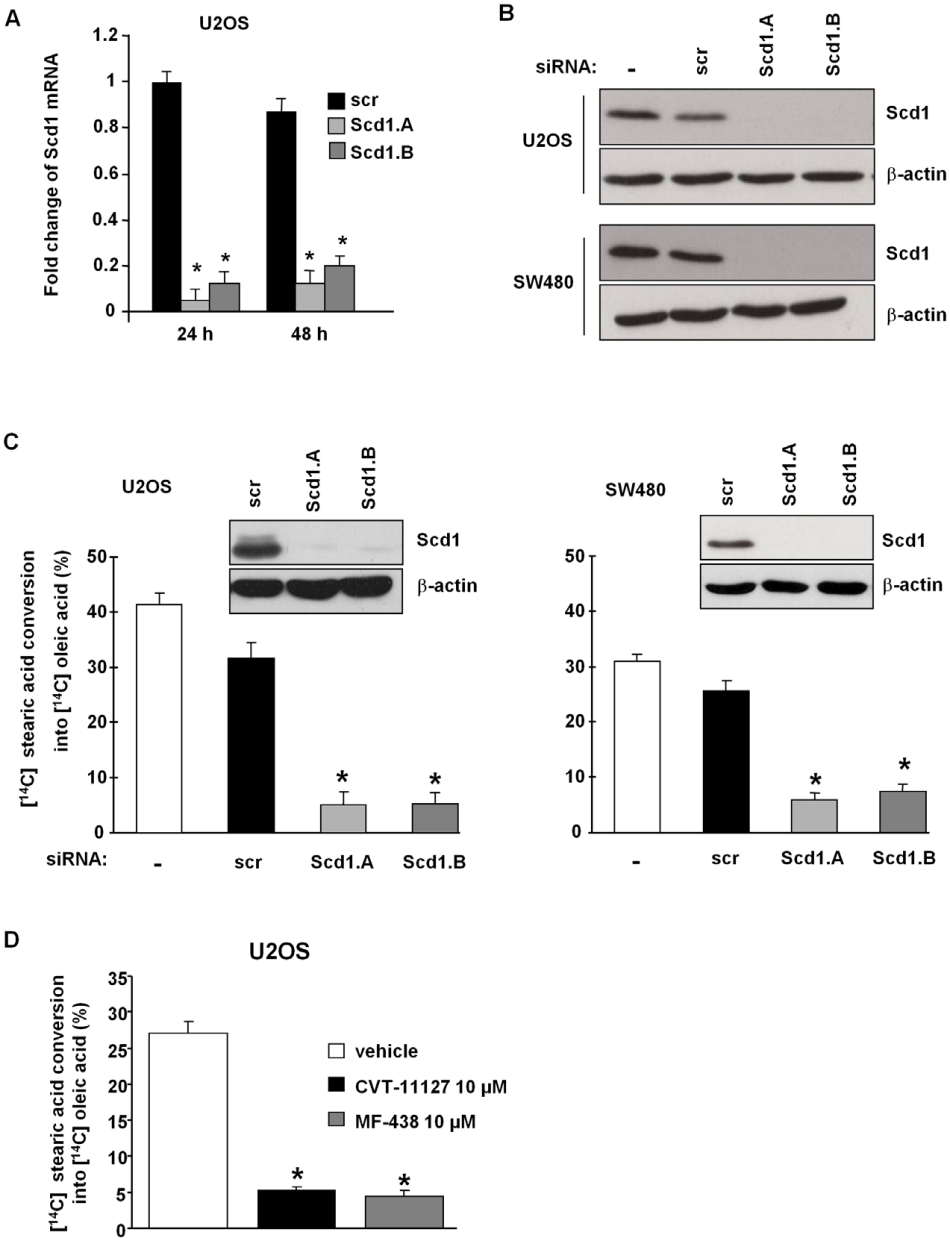
Efficient suppression of Scd1 expression and activity by siRNA targeting Scd1. **A**) U2OS cells were transfected with siRNA control (scr) or with siRNA against Scd1 (Scd1.A and Scd1.B) and collected 24 and 48 h post-transfection for Scd1 mRNA expression by real time RT-PCR. Scd1 mRNA expression was normalized to β-actin expression. Values represent the mean ± SEM relative to Scd1 mRNA expression in scr-treated U2OS cells at 24 h. *, p<0.05 vs. siRNA scr-treated cells by Anova analysis followed by Tuckey test. **B**) U2OS and SW480 cells were treated with oligofectamine (-), siRNA control (scr) or with siRNA against Scd1 (Scd1.A and Scd1.B). Cells were collected 24 h after transfection for Scd1 expression analysis by Western-Blotting. **C**) U2OS and SW480 cells treated 72 h with siRNA were incubated for further 6 h with [^14^C] stearic acid and total lipid extraction was performed. Scd activity was evaluated by HPLC as the rate of [^14^C] stearic acid conversion into [^14^C] oleic acid in cells treated with siRNA for 72 h. Scd activity was expressed as the % ratio of [^14^C] oleic acid to [^14^C] oleic and stearic acids. Values represent the mean ± SEM from at least two separate experiments. *, p<0.05 vs. siRNA scr-treated cells by Anova analysis followed by Tuckey test. A representative expression of Scd1 protein was shown for 72 h of siRNA treatment. D) U2OS cells were exposed to DMSO as vehicle, Scd1 inhibitors (CVT-11127 or MF-438) at 10 µM for 24 h and prepared as above C) for measuring Scd activity. Values represent the mean ± SEM from three experiments. *, p<0.05 vs. vehicle-treated cells by Anova analysis followed by Tuckey test.

In this study we investigated the effect of Scd1 silencing using siRNA in different human cancer cell lines (U2OS, SW480 and HCT116). Cancer cells were transfected with 75 nM siRNA targeting unrelated human mRNA (siRNA scr) and Scd1 mRNA (siRNA Scd1.A and Scd1.B). Both siRNA directed against Scd1 compared to control siRNA (scr) drastically suppressed expression of Scd1 mRNA and protein as soon as 24 h after transfection (Figures 1A and 1B).

As Scd1 catalyzes the conversion of stearic acid into oleic acid, a decrease in oleic acid production would evidence the abolition of Scd1 activity by siRNA targeting this enzyme. In order to address Scd activity after 72 h of Scd1 silencing, we further treated U2OS and SW480 cells for 6 h with radiolabelled [^14^C] stearic acid leading to measure the production of [^14^C] oleic acid in Scd1-deficient cells compared to control scr-treated cells. The incorporation of [^14^C] stearic acid was similar in siRNA scr and Scd1-treated cells (data not shown). Cancer cells transfected by the non-targeting human mRNA siRNA (scr) presented a desaturation rate of 31.49% for U2OS and 25.66% for SW480. In the two cell lines, Scd1 extinction led to a drop-off in oleic acid biosynthesis with a remaining desaturation rate of 5.135% and 5.28% in U2OS treated by siRNA Scd1.A and Scd1.B, respectively, and 5.89% and 7.55%, respectively, in SW480 (Figure 1C). Furthermore, we exposed U2OS cells for 24 h to the Scd1 inhibitors CVT-11127 and MF-438 (10 µM). We obtained similar capability with Scd1 inhibitors than siRNA directed against Scd1 to inhibit the production of oleic acid from stearic acid in U2OS cells (Figure 1D).

Altogether, these results demonstrated a drastic inhibition of Scd activity in siRNA Scd1-treated cells. Moreover, in these cancer cell lines, Scd1 appears as the main enzyme involved in the endogenous production of oleic acid.

### Scd1 extinction promotes apoptosis-cell death

**Figure 2:**
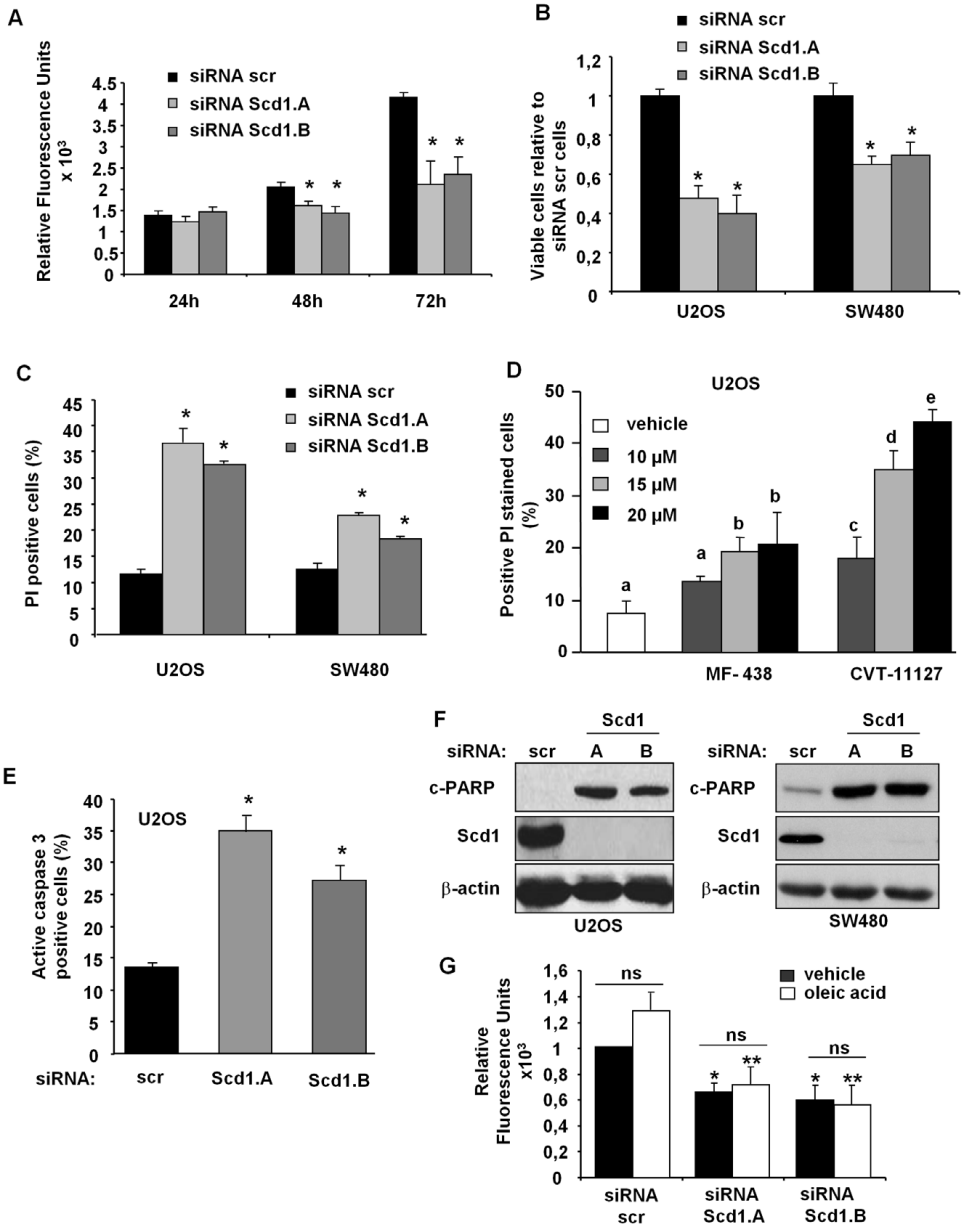
Scd1 knockdown promoted apoptosis-cell death. **A**) U2OS cells were treated with siRNA scr (control) and targeting Scd1 (Scd1.A and Scd1.B), and collected 24 h, 48 h or 72 h post-transfection. Proliferation status was determined by the CyQuant® proliferation assay. Each value is the mean of relative fluorescence units ± SEM of triplicate and representative of three independent experiments. **B**) U2OS and SW480 cells were cultured for 72 h post-siRNA transfection and harvested for trypan blue dye exclusion assay. Values are the mean ± SEM of triplicate and representative of two others independent experiments. **C**) U2OS ad SW480 cells treated 72 h with siRNA were collected and total cell death was analysed by flow cytometry after staining with propidium iodide (PI). Data represent the mean ± SEM of three independent experiments. **D**) U2OS cells were treated for 48 h with Scd1 inhibitors at indicated concentrations and were harvested for propidium iodide staining. Data represent the mean ± SEM from three experiments. **E**) U2OS cells were collected 72 h post-transfection and prepared for caspase 3 activity measurement by flow cytometry as detailed in the materials and methods. Data are shown as fold increase over the control (siRNA scr) and represent the mean ± SEM of two independent experiments. **F**) Whole-cell lysates were prepared 72 h post-transfection with siRNA and PARP cleavage (c-PARP) level was determined by Western-blot. **G**) U2OS cells were treated for 72 h with siRNA control (scr) and targeting Scd1 in absence (vehicle) or presence of 100 µM oleic acid bound to BSA. Cell number was quantified by CyQuant® proliferation assay as previously described. Data are shown as fold change over the vehicle siRNA scr-treated cells and represent the mean of relative fluorescence units ± SEM of triplicate. *, ** p<0.05 vs. siRNA scr-treated vehicle and oleic acid cells, respectively, by Anova analysis followed by Tuckey test.

In order to assess effect of Scd1 knockdown on cell viability, we first determined cell number at 24 h, 48 h and 72 h post-transfection using CyQuant® reagent which quantifies the amount of nucleic acids. As soon as 48 h post-transfection, cell number was significantly less in Scd1-depleted U2OS cells compared to control cells. While relative fluorescence (RF) increased for siRNA scr-treated cells all along the 72 h post-transfection, RF did not significantly change for siRNA Scd1-silenced cells during the time course. We observed that RF was twice fold higher in siRNA scr cells compared to siRNA Scd1-depleted U2OS 72 h post-transfection indicating proliferation inhibition or cell death induction in Scd1-ablated cells (Figure 2A). Then, we demonstrated by trypan blue exclusion cell count 72 h after transfection of siRNA that Scd1 knockdown led to a diminution of cell viability both in U2OS and SW480 cells but much more drastically in U2OS cells (Figure 2B). More than 30% of Scd1 siRNA-treated U2OS and approximately 20% of Scd1 siRNA-treated SW480 were positive for PI representing an increase of three and two folds compared to siRNA scr-treated U2OS and SW480 cells, respectively (Figure 2C). Inhibition of Scd1 activity by both compounds (CVT-11127 and MF-438) also led to increase cell death at 48 h in a dose-response manner (Figure 2D). However, we found that these compounds differently affected cell viability with CVT 11127 more potent for cell death induction than MF-438 in U2OS. Moreover, we showed that Scd1 depletion induced activation of apoptosis as shown by high-level induction of caspase 3 activity and PARP cleavage (Figures 2E and 2F).

We then postulated that cell death was triggered by reduction of oleic acid cell content. Thus, we undertook to supplement siRNA Scd1-depleted cells with 100 µM oleic acid in order to evaluate the capability of exogenous supplementation to reverse cell death. Figure 2G evidenced that exposure of Scd1-deficient U2OS cells to exogenous oleic acid did not change the rate of cytotoxicity.

### Inhibition of Scd1 expression activates partially unfolded protein response

**Figure 3:**
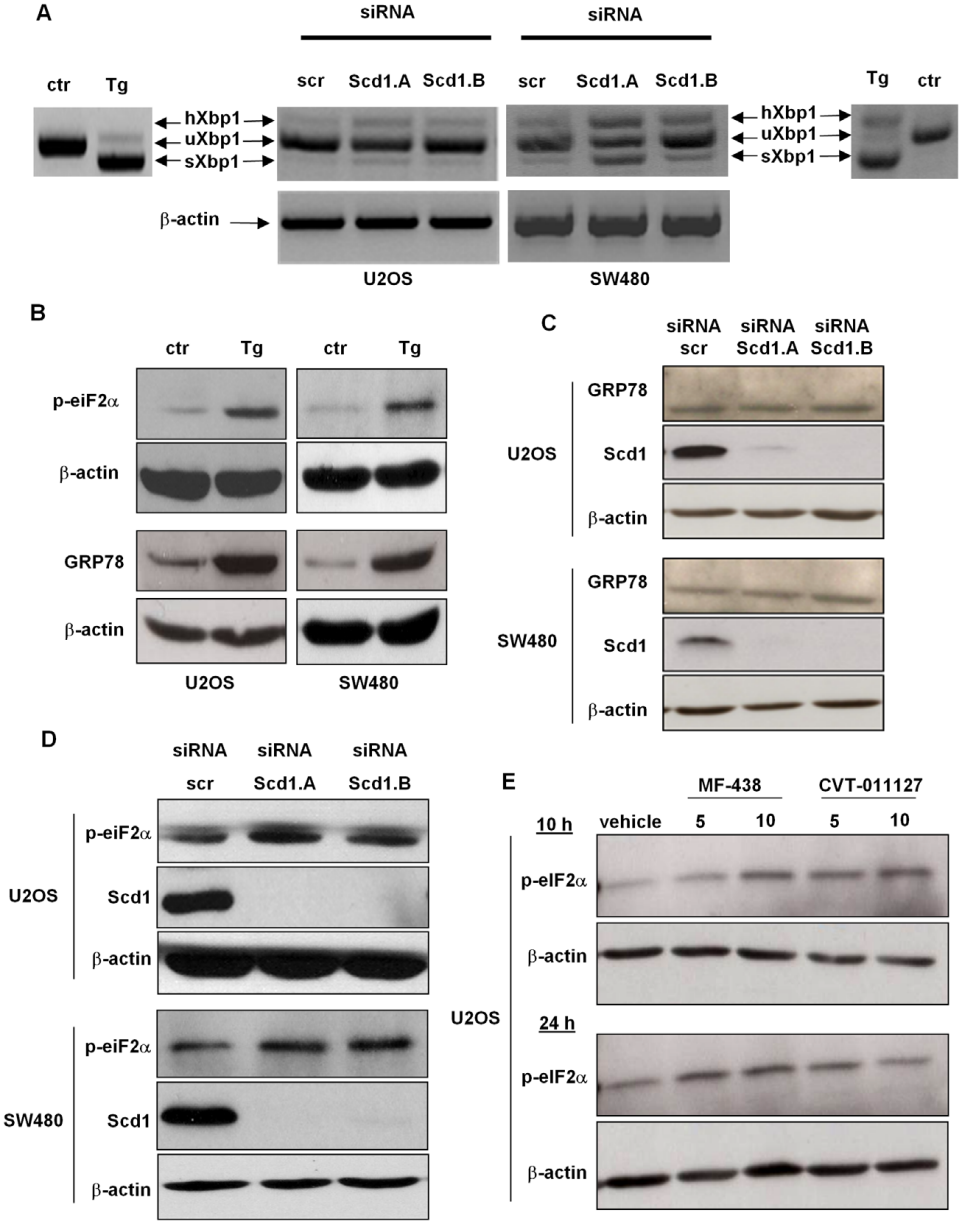
Inhibition of Scd1 partially induced UPR markers. U2OS and SW480 cells were treated with siRNA control (scr) and siRNA against Scd1 for 72h. **A**) Samples were prepared for mRNA analyses of Xbp1 processing by semi-quantitative RT-PCR. The PCR products were run on a 3% agarose gel and the spliced Xbp1 (sXbp1), unspliced Xbp1 (uXbp1) and hybrid Xbp1 (hXbp1) mRNA species were observed in siRNA-treated cells (ctr) and thapsigargine-treated cells (Tg) as positive control of UPR activation. **B**) Total protein lysates were prepared from untreated and thapsigargin-treated cells (Tg, 0.2 µM, 16 h) and analysed for eiF2α phosphorylation and GRP78 up-regulation by western-blotting. **C and D**) Scd1-depleted U2OS and SW480 cells were prepared as in 3B) and analysed by western-blotting for Scd1, GRP78 and phospho-eiF2α expression. **E**) U2OS cells were treated with 5 and 10 µM of Scd1 inhibitors (MF-438 and CVT-11127) and were collected after 10 h and 24 h of treatment for phospho-eiF2α expression analysis by western-blotting. Blots are representative of at least two independent experiments.

Perturbations of ER homeostasis lead to ER stress by UPR activation that could trigger cell death. In order to monitor activation of the UPR pathway, we investigated the expression level of GRP78, phospho-eIF2α and unconventional splicing of Xbp1 mRNA. U2OS and SW480 cells have the functional machinery to respond to thapsigargin-induced ER stress, as we observed splicing of Xbp1, up-regulation of GRP78 and phospho-eIF2α expression (Figures 3A and B). Then, we analyzed these ER stress markers in Scd1-deficient cells. Abolition of Scd1 in U2OS and SW480 cells led to a partial processing of Xbp1 mRNA: spliced- and hybrid-Xbp1 (s- and h-Xbp1) mRNA species were increased in Scd1-deficient cells indicating activation of IRE1α arm in both cell lines, in a more pronounced manner in SW480 cells (Figure 3A). Translation of s-Xbp1 produces the functional Xbp1 transcription factor, which participates to a transcription program in order to first re-establish ER function and cell survival. The chaperone GRP78 also regulates the pro-survival pathway during ER stress through its up-regulation. However, we were not able to observe such regulation in U2OS and SW480 cells silenced for Scd1 48 h post-transfection (Figure 3C). We did not observe any change in mRNA and protein level for GRP78 expression at different post-transfection time (24, 48 and 72 h, data not shown). PERK also belongs to UPR and its activation induces phosphorylation of eIF2α triggering repression of general translation. We observed at 48 h post-transfection that cells depleted in Scd1 expressed higher amount of phospho-eIF2α compared to control cells suggesting activation of the PERK arm (Figure 3D). In order to assign phosphorylation of eiF2αto Scd1 extinction and not to an artefact due to PKR activation by siRNA transfection, we analysed phospho-eIF2α level in U2OS treated with 5 and 10 µM of Scd1 inhibitor (CVT-11127 and MF-438) for 10 and 24 h. We observed increase of p-eIF2α expression as soon as 10 h for both inhibitors demonstrating that phosphorylation of eIF2α was induced by extinction of Scd1 activity (Figure 3E).

### CHOP participates to Scd1 depletion-induced cell death

**Figure 4:**
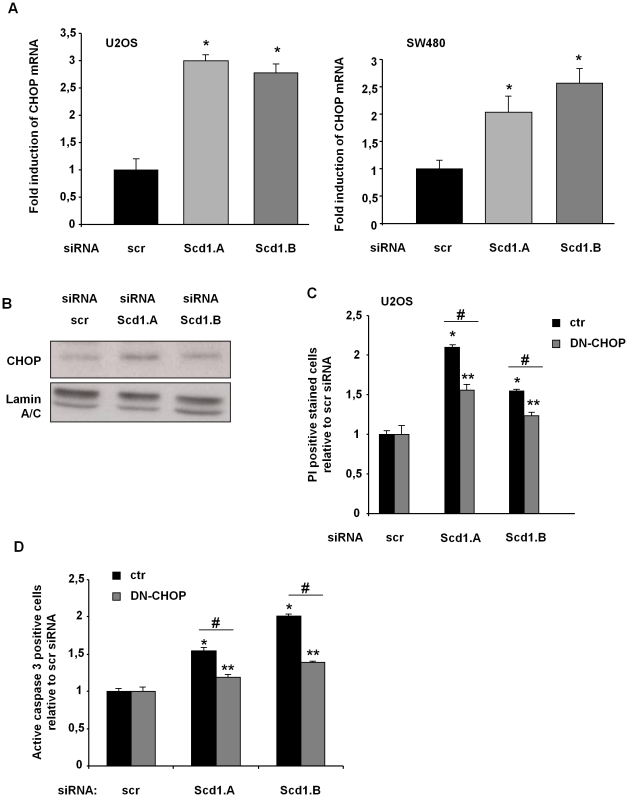
Inhibition of CHOP activity reduced siRNA Scd1-mediated U2OS cell death. **A**) U2OS and SW480 cells were treated with siRNA control (scr) and siRNA against Scd1 for 72h. Total RNA was isolated and CHOP mRNA expression normalized to β-actin mRNA expression was quantified by real time RT-PCR. Results were represented as mean fold induction ± SEM relative to siRNA scr-treated cells from at least three independent experiments. *, p<0.05 vs. siRNA scr-treated cells by Anova analysis followed by Tuckey test. **B**) Nuclear extracts from U2OS were prepared 72 h after siRNA addition. CHOP expression was analysed by western-blotting and lamin A/C was loading control of nuclear extract samples. **C**) U2OS cells were transiently transfected with empty (ctr) or dominant-negative CHOP (DN-CHOP) expression vector and selected for three days in G418. Resistant cells transfected by ctr or DN-CHOP construct were treated by siRNA control scr or against Scd1 (Scd1.A and Scd1.B) for 72 h and harvested for propidium iodide staining analysis by flow cytometry. Values were shown as fold increase over the controls (siRNA scr) and represent the mean ± SEM of two independent experiments. *, **, p<0.05 vs. siRNA scr-treated ctr and DN-CHOP cells, respectively, by Anova analysis followed by Tuckey test. #, p<0.05 by Anova analysis followed by Tuckey test. **D**) U2OS cells were prepared as C) and harvested for active caspase 3 analysis by flow cytometry. Values were shown as fold increase over the controls (siRNA scr) and represent the mean ± SEM of three independent experiments. *, **, p<0.05 vs. siRNA scr-treated ctr and DN-CHOP cells, respectively, by Anova analysis followed by Tuckey test. #, p<0.05 by Anova analysis followed by Tuckey test.

In ER stress-mediated apoptosis, CHOP expression increases and appears as an essential effector of this cell death program. We first addressed evaluation of CHOP expression in cancer cells treated with siRNA scr or against Scd1. We observed an increase of CHOP mRNA and protein expression in cells that lost Scd1 expression compared to control cells (Figures 4A and 4B). To ascertain the role of CHOP in cell death induction, we transiently transfected empty vector (ctr) or dominant-negative form of CHOP (DN-CHOP) in U2OS cells. DN-CHOP construct harbours mutations in the leucine zipper domain (L134A/L141A) that prevents its transcriptional activity [37]. We showed by PI staining that DN-CHOP overexpression reduced cytotoxicity induced by Scd1 inhibition compared to the control-transfected cells (ctr) (Figure 4C). Moreover, we estimated the effect of enforced expression of DN-CHOP on active caspase 3 induction in Scd1-silenced U2OS. We showed a decrease of caspase 3 activation in Scd1 knockdown-U2OS cells expressing DN-CHOP and evidenced a protective effect of DN-CHOP against apoptosis induced by Scd1 depletion (Figure 4D).

### CHOP extinction partially alleviates Scd1 depletion-induced apoptosis

**Figure 5:**
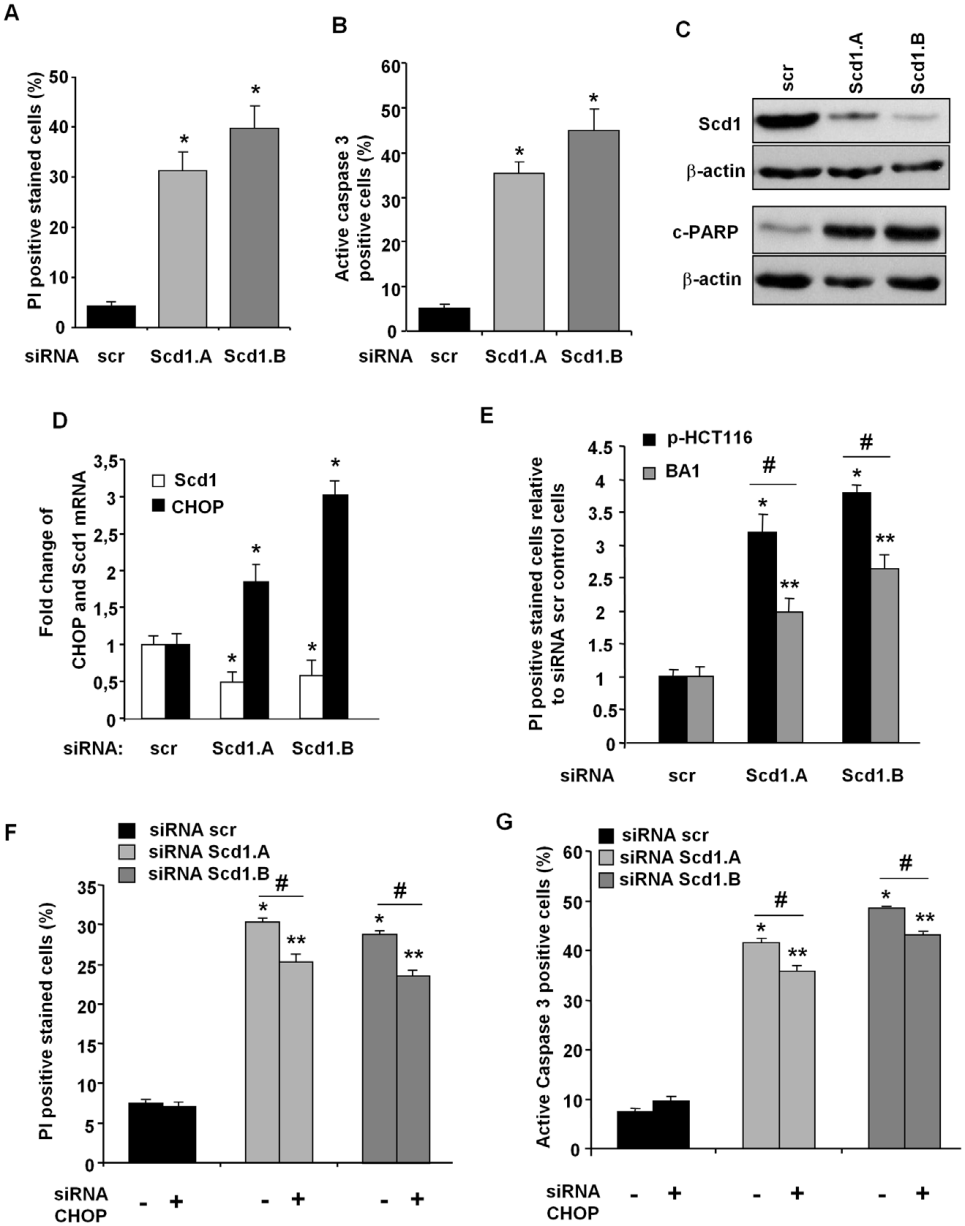
Extinction of CHOP expression reduced Scd1 knockdown-mediated HCT116 apoptosis. **A**) HCT116 cells treated 72 h with siRNA control (scr) or targeting Scd1 (Scd1.A and Scd1.B) were collected and total cell death was analysed by flow cytometry after staining with propidium iodide. Results were the mean ± SEM of three experiments performed in triplicate. **B**) HCT116 cells treated by siRNA were collected 72 h post-transfection and prepared for caspase 3 activity measurement by flow cytometry as detailed in the materials and methods. Data represent the mean ± SEM of three independent experiments. **C**) HCT116 were treated as in A) and harvested for analysis of Scd1 and PARP-cleavage expression by Western-blotting. **D**) HCT116 were treated as in A) and harvested for total RNA purification. CHOP and Scd1 mRNA expression was analyzed by real time RT-PCR after normalization to β-actin. All experiments represent at least two repetitions in triplicate. **E**) HCT116 cells were stably transfected with antisense human CHOP cDNA construct and its empty control vector. BA1 and p-HCT116 are HCT116 clones with antisense CHOP construct and control vector, respectively. Cells were silenced by siRNA Scd1.A and Scd1.B for 72 h, and harvested for total cell death analysis by flow cytometry after staining with propidium iodide. Results were the mean of fold change for PI positive cells relative to the corresponding siRNA scr HCT116 cells ± SEM of one representative experiment from three experiments performed in triplicate. *, **, p<0.05 vs. siRNA scr-treated p-HCT116 and BA1 cells, respectively, by Anova analysis followed by Tuckey test. #, p<0.05 by Anova analysis followed by Tuckey test. **F**) and **G**) HCT116 cells were transfected with siRNA scr, Scd1.A or Scd1.B at 75 nM, and either with 25 nM siRNA scr (-) or siRNA CHOP. Cells were collected at 72 h post-transfection for PI staining and active caspase 3 analyses by flow cytometry. Results were the mean ± SEM of one representative experiment from two independent experiments performed in triplicate. *, **, p<0.05 vs. corresponding siRNA scr-treated HCT116 cells by Anova analysis followed by Tuckey test. #, p<0.05 by Anova analysis followed by Tuckey test.

The protection observed by CHOP inhibition against Scd1 depletion-mediated U2OS cell death has also been evaluated in HCT116 colon tumor cell line. In this aim, we used transitory CHOP knockdown-HCT116 and BA1 cells that are HCT116 cells stably transfected with antisense human CHOP cDNA construct [38]. Depletion of Scd1 with siRNA in HCT116 induced more than 30% of cell death as attested by PI staining (Figure 5A). We also showed at 72 h that extinction of Scd1 activity by siRNA and inhibitors (data not shown) induced apoptosis in HCT116 evidenced by active caspase 3 or PARP-cleavage detection (Figures 5B and 5C). In those cells, we also found that abolition of Scd1 expression led to up-regulation of CHOP mRNA expression as already observed for U2OS and SW480 cells (Figure 5D). Furthermore, we confirmed as in U2OS that reduction of CHOP expression partially alleviated cytotoxicity induced by Scd1 silencing. Indeed, we observed a diminution of PI staining in BA1 cells compared to their parental HCT116 (p-HCT116) counterparts when Scd1 expression was abolished (Figure 5E). Moreover, the protection against Scd1 silencing-mediated cell death induced by CHOP extinction seemed to be specific and not a protection against all pro-apoptotic factors. Indeed, extinction of CHOP did not modify apoptosis induction by etoposide in DN-CHOP U2OS or BA1 compared to their respective control cells (data not shown). We have also undertaken to evaluate effect of CHOP extinction by siRNA treatment on apoptosis induced by Scd1 abolition. For this purpose, we performed co-treatment of HCT116 cells with siRNA control (scr) or directed against Scd1 (Scd1.A and Scd1.B), and with siRNA CHOP or control (−).We validated CHOP siRNA in HCT116 cells and showed that CHOP siRNA did not modify Scd1 mRNA extinction level induced by Scd1 siRNAs (data not shown). In order to estimate CHOP function in Scd1-induced apoptosis, we then performed siRNA co-transfection. We observed 72 h after transfection that CHOP silencing protected HCT116 against apoptosis-cell death induced by Scd1 extinction (Figures 5F and 5G).

### Scd1 depletion did not affect the viability of non cancer cells

**Figure 6:**
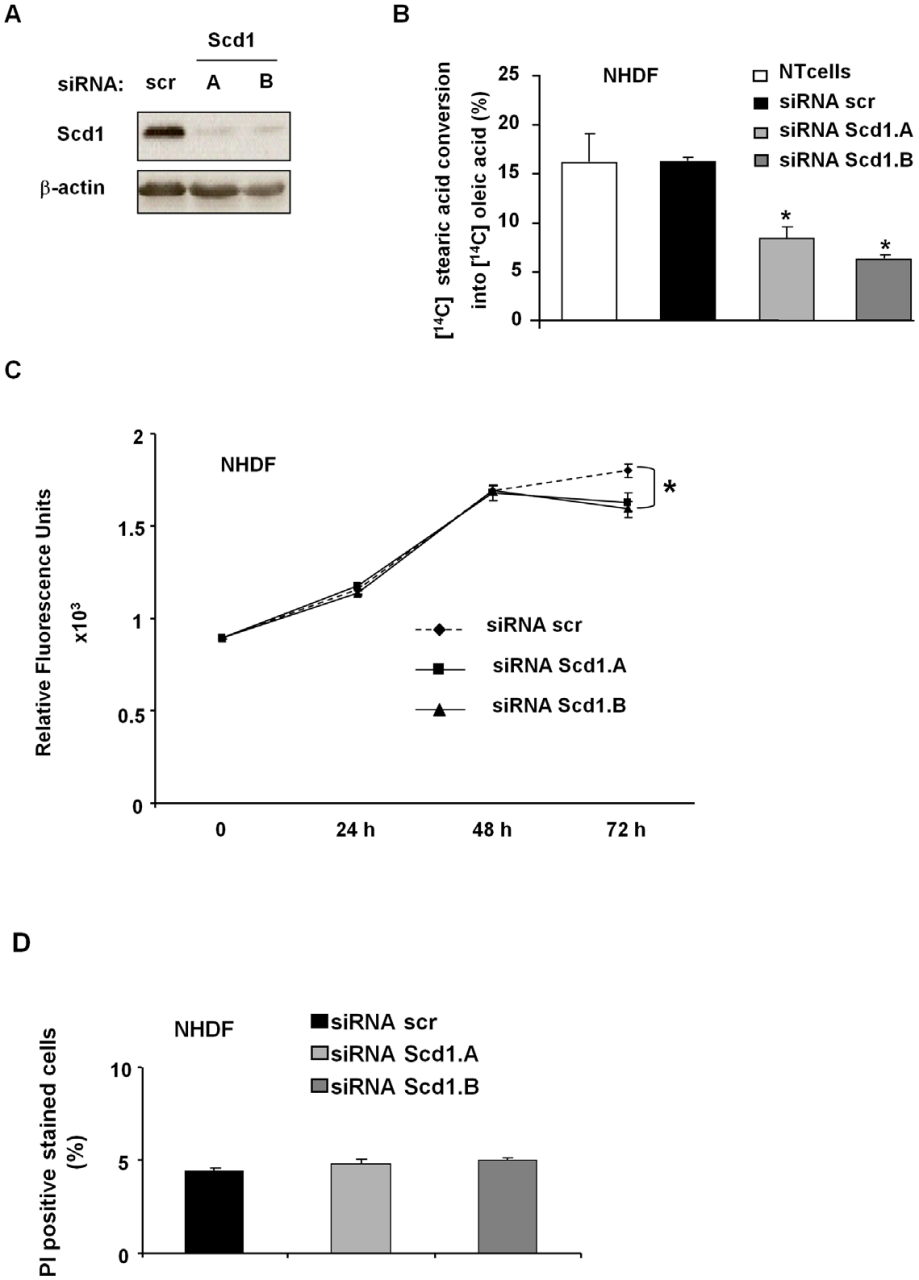
Scd1 depletion did not affect viability of non cancer cells. Normal human dermal fibroblasts (NHDF) were transfected with siRNA control (scr) and siRNA targeting Scd1 (Scd1.A and Scd1.B). NHDF cells were harvested after 72 h siRNA treatment and harvested for further analyses. **A**) Total proteins were prepared for Scd1 expression analysis by western-blotting. **B**) NHDF treated 72 h with siRNA were incubated for further 6 h with [^14^C] stearic acid and total lipid extraction was carried out. Conversion of [^14^C] stearic acid into oleic acid was performed by HPLC. Scd activity was expressed as the % ratio of [^14^C] oleic acid to [^14^C] oleic and stearic acids. Values represent the mean ± SEM for at least two separate experiments. **C**) Proliferation status of siRNA-treated NHDF was determined at indicated time by the CyQuant® proliferation assay. Each value is the mean of relative fluorescence units ± SEM of three independent experiments. **D**) NHDF were collected 72 h post-transfection and total cell death analysis was performed by flow cytometry after staining with propidium iodide. Results were the mean ± SEM of PI positive cells (%) of one representative from two experiments performed in triplicate. *, p<0.05 vs. siRNA scr-treated cells by Anova analysis followed by Tuckey test.

Cancer cells were sensitive to Scd1 depletion-mediated cell death.We were then interested in analysing the potential effect of an absence of *de novo* MUFA synthesis in non cancer cells. In this aim, we evaluated impact of Scd1 inhibition using siRNA on normal human dermal fibroblasts (NHDF). These cells have a basal desaturation rate about 15% lower than U2OS and SW480 cells as shown previously in Figure 1C. Their treatment with siRNA Scd1.A or Scd1.B led to a reduction of Scd activity to reach a remaining activity of 8.3% and 6.3%, respectively (Figure 6B). The residual Scd activity was similar to the one obtained for cancer cells treated with the siRNA Scd1. As shown above, ablation of Scd activity in cancer cells led to cytotoxicity whereas depletion of Scd1 expression did not induce cell death in NHDF but reduced very slightly their cell number (Figures 6C and 6D). Then, in non cancer cells, Scd1 abrogation might block proliferation without affecting cell viability.

## Discussion

In the present study, we demonstrated that Scd1 expression silencing led to induction of UPR markers (Xbp1 splicing, p-eIF2α and CHOP) and CHOP-dependent cell death in cancer cells. We also showed that abrogation of de novo MUFA synthesis pathway by extinction of its rate-limiting enzyme Scd1 altered viability of cancer cells without changing the survival of non cancer cells. This data suggests different needs in de novo MUFA synthesis for normal and tumoral cells. Nevertheless, exogenous addition of oleic acid, the major MUFA product of Scd1 activity, did not prevent cell death of cancer cells in which endogenous MUFA biosynthesis was suppressed.

In this report, we agree with previous reports describing that Scd1 extinction led to cell death by apoptosis in different types of cancer cells [22], [25], [27], [39]. Indeed, we observed induction of caspase 3 activity and PARP-cleavage (Figures 2D et 2E) in Scd1-depleted cells.

We also evidenced in this work that normal and cancer cells did not respond in the same manner to the prevention of MUFA synthesis by Scd1 extinction. Indeed, while cancer cells were killed by Scd1 depletion, non cancer cells were still alive. However, we observed a slight decrease in cell number in Scd1-treated NHDF that a block or a slower rate of proliferation could explain. We can then hypothesise that viability of non cancer cells remained unaffected due to the fact that they do not require such a rapid and high MUFA synthesis. Indeed, they proliferate at lower rate (Figure 6C) and preferentially sustained new membrane synthesis from exogenous fatty acids up-take whereas cancer cells proliferate at higher rate and need de novo fatty acid synthesis [10].

We and others showed that abolition of Scd1 activity decreased de novo MUFA production (Figure 1C) and in consequence increased SFA content (e.g. palmitic and stearic acids) [22], [26], [27], [40]. Induction of lipotoxicity by SFA in cancer and non cancer cells has been previously described, and that the level of Scd1 expression controlled SFA effect since overexpression of Scd1 reduced SFA-mediated cell death when a down-regulation increased it [27], [29], [39], [41], [42], [43]. Among MUFA, oleic acid is the most represented and has been characterised by cytoprotective and proliferative actions. Its addition promotes tumoral cell proliferation that could be obtained through down-regulation of the tumor suppressor Pten as described for hepatocarcinoma cells but also prevents palmitate-stimulated apoptosis [29], [41], [44]. As already reported for SV40-tranformed lung fibroblasts lacking Scd1 expression, we were not able to observe any rescue of siRNA Scd1-induced U2OS cell death (Figure 2F) [39]. This observation is in discrepancy with recent results indicating that oleic acid could reverse the impairing effects of Scd1 depletion on proliferation and survival in Hela and H460 tumor cells [27], [45]. A possible mechanism of oleic acid protection is the increase of triglyceride production that could incorporate SFA excess into triglycerides and protects cells from apoptosis [45], [46]. The observed discrepancy could be due to a different ability of Scd1-depleted U2OS cells placed under oleic acid supplementation to channel SFA into triglyceride pool.

Scd1 controls the de novo synthesis of MUFA, and, more globally appears as a key regulator of lipid metabolism. In order to maintain their high proliferating rate, cancer cells require an elevated production of cellular membrane components membrane such as cholesterol and phospholipids [47], [48]. Interfering with the phospholipid Kennedy's pathway is described to block phospholipid synthesis and to induce apoptosis [30], [49], [50]. Thus, extinction of Scd1 activity decreases de novo phospholipid and neutral lipid synthesis, and cell death obtained by Scd1 depletion in different cancer cells could be related to this change [39].

On the other hand, the phospholipid remodelling which represented the decrease of unsaturated fatty acid incorporation consecutive to Scd1 depletion played a predominant role in cell survival by triggering cell death [27].

Thus, both phospholipid synthesis and remodelling pathways seemed to be involved in Scd1 inhibition-mediated cell death. Additionally, changes in phospholipid metabolism can induce ER stress response that could explain its activation in Scd1-depleted cells [30].

Indeed, we clearly showed, in the present study, a peculiar ER stress activation since GRP78 expression was not modified. GRP78, a stress-inducible chaperone localized in the ER, is a key component of the UPR required to alleviate ER stress, maintain ER function and protect cells against apoptosis [51]. Lack of anti-apoptotic GRP78 induction could contribute to cell death in maintaining the three transducers of ER stress (PERK, IRE1α and ATF6) under an activated status and down-regulation of GRP78 is known to sensitize tumor cells to apoptosis induced by current anti-cancer therapies [52], [53], [54]. The observation concerning GRP78 expression in Scd1-depleted cells was not related to the inability of U2OS and SW480 cells to increase GRP78 expression in ER stress response since thapsigargin-treated cells overexpressed GRP78 protein. Few reports already mentioned such a lack of GRP78 induction during ER stress. Among them, inhibition of phosphatidylcholine synthesis, treatment with palmitic acid or trans 10, cis 12-conjugated linoleic acid triggered ER stress and cell death without affecting GRP78 content [30], [55], [56].

We pointed out in the present study that the extinction of de novo MUFA synthesis via Scd1 knockdown led to the most characteristic UPR induction with activation of PERK and IRE1α arms. Indeed, Scd1 depletion-induced ER stress triggered Xbp1 mRNA splicing (Figure 3A) demonstrating activation of IRE1α arm as previously described [57]. Xbp1 processing, even partial, highlighted IRE1α activation through dissociation from GRP78 in response to Scd1 inhibition. This data was confirmed by the increased amount of phospho-eIF2αthat we observed in Scd1-depleted cells indicating activation of PERK via its dissociation from GRP78 (Figure 3D). Phosphorylation of eIF2α leads to inhibition of global translation, concomitant with selective translation of a subset of transcripts including the ATF4 transcription factor, to first cope with ER stress and restore ER function [33].

However, activation of PERK arm was also described as part of a pathway involved in cell death [58]. Thus, Ariyama et al. showed that Scd1 extinction triggered PERK activation and confirmed that PERK arm could be a pro-apoptotic signal in preventing Scd1 extinction-mediated cell death by down-regulating PERK expression [27]. Our observation on phosphorylation of eIF2α highlighted data about PERK activation in Scd1-ablated cells but others stress-inducible eIF2α kinases (e.g. GCN2, PKR) could also induce this post-translational modification and the associated cell death [59].

The phosphorylation of eIF2α triggers ATF4 mRNA translation, which is known to up-regulate the expression of the pro-apoptotic transcription factor CHOP in cellular stress context [33]. CHOP could trigger apoptosis at least through transcription induction of pro-apoptotic genes (e.g. BIM) and transcription repression of anti-apoptotic ones (e.g. Bcl-2) [34], [60]. Here, we confirmed that Scd1 knockdown enhanced CHOP expression and activated apoptosis evidenced by PARP-cleavage and active caspase 3 production. Although CHOP inactivation led to a decrease of apoptosis induction in Scd1-depleted cells, we were not able to fully restore the viability suggesting that other pathways are probably involved in cell death induced by Scd1 inactivation. During severe ER stress, apoptosis at least appears through CHOP induction. However, caspase 12 (in rodents) and JNK pathway are also involved in ER stress-mediated apoptosis and might participate to Scd1 extinction-induced apoptosis [61].

In conclusion, our results provided evidences that Scd1 extinction induced UPR and cell death in cancer cells through CHOP activation. Thus, this study highlights that components of de novo MUFA synthesis and UPR pathway represent promising targets for cancer therapy.

## Materials and Methods

### Materials

U2OS human osteosarcoma cancer cells, SW480 human colorectal adenocarcinoma cancer cells were obtained from American Type Culture Collection (Rockville, MD). BA1 cells stably expressing human CHOP antisense cDNA construct and their parental HCT116 cells counterparts were graciously given by Dr. Jesse Martinez. Normal human dermal fibroblasts (NHDF) were obtained from Invitrogen (Cergy-Pontoise, France). Cell culture supplies were from Dutscher (France). Anti-Scd1, anti-CHOP/GADD153, anti-GRP78 were purchased from Santa-Cruz biotechnologies, anti-peIF2α, anti-cleaved PARP, anti-lamin A/C from Cell Signaling Technology and anti-β-actin from Sigma. Oleic acid was obtained from NuCheck Prep (Elysian, MN) and [^14^C] stearic acid from Perkin-Elmer (Courtaboeuf, France). Scd1 inhibitors CVT-11127 and MF-438 were generous gift from Gilead Sciences, Inc. and Merck Frosst, respectively [62], [63].

### Cell culture and transfections

U2OS, SW480, HCT116 cells were maintained in DMEM supplemented with 10% heat inactivated foetal bovine serum (FBS), penicillin (100 U/ml), streptomycin (100 µg/ml), L-glutamine (2 mM). NHDF were cultivated in MEM106 (Invitrogen) supplemented with LSGS (Invitrogen), 10% heat inactivated FBS, penicillin (100 U/ml), streptomycin (100 µg/ml), L-glutamine (2 mM). For experiments of fatty acid supplementation, oleic acid bound to BSA (ratio 4∶1) was used at 100 µM.

Small interfering RNAs (siRNA) were synthesized by Dharmacon Research Inc. (Lafayette, CO) and were transfected into cells with Oligofectamine (Invitrogen) according to the manufacturer's recommendations. Transfected cells were collected at indicated post-transfection time. siRNA control (scr) targets unrelated human mRNA CUUACGCUGAGUACUUCG; siRNAs against Scd1 target GAUAUGCUGUGGUGCUUAA (Scd1A) and GAGAUAAGUUGGAGACGAU (Scd1.B); siRNA CHOP targets CAAUUGUUCAUGCUUGGUG sequence [25].

Scd1 inhibitors CVT-11127 (Gilead Sciences, Inc.) and MF-438 (Merck Frosst) were dissolved in DMSO and used at indicated concentrations [62], [63].

U2OS were transiently transfected with empty or dominant-negative CHOP (DN-CHOP) expression vector with lipofectamine 2000 (Invitrogen) and selected next day in G418 (400 µg/ml) for three days. Then, cells were trypsinised and seeded at 130 000 cells/well in 6-well plate. Next day, siRNA transfection was performed as described above.

### Cell proliferation, viability and death analyses

Cell proliferation analysis was carried out using CyQuant® NF cell proliferation assay (Invitrogen). Adherent cells were washed and frozen at –80°C. Cells were thawed and treated with CyQuant GR dye, which binds stoichiometrically to nucleic acids and, thus, measures cell number. Fluorescence quantification was measured using a microtiter plate fluorimeter (VICTOR^3^V™, PerkinElmer Life Sciences Inc., Wellesley, MA) with excitation at 485 nm and detection at 535 nm.

Cell viability was evaluated by trypan blue exclusion assay using phase contrast microscopy at indicated time after siRNA treatment. Cells that exclude trypan blue dye are considered to be viable.

Total cell death was determined by propidium iodide staining (PI) using flow cytometry (FACSCalibur, Becton Dickinson). Cells were collected 72 h after siRNA treatment. PI (1 µg/ml) was added to cells and positive PI staining cells were considered as dead cells.

Caspase 3 activity was determined using CaspGLOW Fluorescein Caspase-3 Staining Kit by flow cytometry. The assay utilizes the caspase-3 inhibitor, DEVD-FMK, conjugated to FITC (FITC-DEVD-FMK) that irreversibly binds to activated caspase-3 in apoptotic cells.

### RNA purification and RT-PCR

Total RNA was purified using RNEasy kit (Qiagen, Germany). Reverse transcription was performed with 1 µg RNA using iScript cDNA Synthesis Kit (Bio-Rad).

For analysis of Xbp1 mRNA splicing, semi-quantitative PCR analysis was performed with Gotaq green master mix (Promega) and the following primers were used:

5′-AAACAGAGTAGCAGCTCAGACTGC-3′ and 5′-TCCTTCTGGGTAGACCTCTGGGAG-3′; and β-actin: 5′-ATGATATCGCCGCGCTCGTCGTC-3′ and antisense 5′-AGGTCCCGG CCAGCCAGGTCCAG-3′.

RNA expression level was quantified by real-time PCR using iQ SYBR Green supermix (Bio-Rad) using a Bio-Rad iCycler iQ with the following primers:

CHOP: sense 5′-ACA CAG ATG AAA ATG GGG GTA CCT-3′ and antisense 5′-AGA AGC AGG ATC AAG AGT GGT-3′; Scd1: sense 5′-TTC AGA AAC ACA TGC TGA TCC TCA TAA TTC CC-3′ and antisense 5′-ATT AAG CAC CAC AGC ATA TCG CAA GAA AGT CTG-3′; β-actin: sense 5′- CTG GTG CCT GGG GCG-3′ antisense 5′-AGC CTC GCC TTT GCC GA–3′.

### Western Blotting

For whole-cell extracts, cells were washed with ice-cold PBS and lysed in ice-cold triton buffer (Tris-HCL 20 mM pH 7.4, Nacl 150 mM, EDTA 0.5 mM, EGTA 0.5 mM, Triton X-100 1%), containing protease and phosphatase inhibitor cocktails (Sigma cocktails I and II) for 15 min on ice. We cleared protein lysate at 10 000 g, 10 min at 4°C. Thirty micrograms of total proteins were loaded for SDS-PAGE electrophoresis and transferred onto a nitrocellulose membrane.

For nuclear extracts, cells were washed with ice-cold PBS and harvested in lysis buffer (10 mM HEPES, pH 7.9, 50 mM NaCl, 0.1 mM EDTA, 500 mM sucrose, 0.5% Triton X-100 with protease inhibitors as described above. After swelling for 10 min on ice, cytosolic fraction was obtained by centrifugation at 2000× *g* for 10 min at 4°C. The pellet was resuspended in nuclei lysis buffer (10 mM HEPES, pH 7.9, 500 mM NaCl, 0.1 mM EDTA, 0.1 mM EGTA, 0.1% NP-40 with protease inhibitors) and incubated on ice for 30 min with vortexing every 5 min. The suspension was centrifuged at 24,000× *g* for 15 min at 4°C and the resulting supernatant was the nuclear extract.

Immunoblotting was performed with antibodies raised against phospho-eIF2α (1/1000), anti-Scd1 (1/500), anti-cleaved PARP (1/1000), anti-GRP78 (1/200), anti-CHOP/GADD153 (1/200), anti-lamin A/C (1/1000) and anti-β-actin (1/4000). Horseradish peroxidase-conjugated secondary antibodies were used at 1/5000.

### Determination of Scd activity

We evaluated Scd activity by measuring the conversion of [^14^C] stearic acid into [^14^C] oleic acid after siRNA (72 h) or Scd1 inhibitor (24 h) treatment. Cells were incubated with 3 µM (0,25 µCi/dish of [^14^C] stearic acid) for 6 h at 37°C in 5% CO2 incubator. Cells were collected and total lipids were extracted according to Bligh and Dyer method. Lipids were saponified and esterified. Radiolabelled fatty acid methyl esters were separated by RP-HPLC and detected on line by a radioisotope detector (Packard Flow Scintillation Analyser, PerkinElmer Life Sciences Inc., Wellesley, MA). The [^14^C] oleic acid/([^14^C] oleic and stearic acids) ratio was determined as Scd activity.

### Statistical analysis

Results are presented as means ± standard error mean (SEM). Statistical significance of results was determined by OneWay Anova analysis followed by Tuckey HSD test. Values of p<0.05 were considered significant.
